# Impact of 2020 SARS-CoV-2 outbreak on telemedicine management of cardiovascular disease in Italy

**DOI:** 10.1007/s11739-020-02564-0

**Published:** 2020-12-08

**Authors:** Giuseppe Molinari, Natale Daniele Brunetti, Savina Nodari, Martina Molinari, Giampietro Spagna, Mariangela Ioakim, Giovanni Migliore, Vitangelo Dattoli, Ottavio Di Cillo

**Affiliations:** 1 “Telemedico Srl” Telecardiology Center, Genova, Italy; 2 Department of Medical and Surgical Sciences, University of Foggia, Foggia, Italy; 3 Department of Medical and Surgical Specialities, Radiological Sciences and Public Health, University of Brescia, Brescia, Italy; 4 Cardiocalm Srl, Montichiari, BS Italy; 5 Policlinico Riuniti, Foggia, Italy; 6 Policlinico Bari, Bari, Italy; 7 Policlinico Riuniti, Foggia, Italy; 8 Agenzia Regionale per la Salute e il Sociale, Bari, Italy

**Keywords:** Covid-19, Telemedicine, Cardiovascular disease

## Abstract

The Covid-19 pandemic affected large part of Italy since February 2020; we, therefore, aimed to assess the impact of 2020 SARS-CoV-2 outbreak on telemedicine management of cardiovascular disease (CVD) in Italy. We analyzed data from three telemedicine dispatch centers, one located in Genoa, serving private clients (pharmacies, general practitioners), one in Brescia, serving pharmacies, and one in Bari, serving regional public STEMI network and emergency medical service in Apulia (4 million inhabitants). Demographic data and principal electrocardiogram diagnosis were collected and analyzed. Records from the time interval March 1, 2020 and April 1, 2020 were compared with the corresponding period in 2019. The comparative analysis of data shows a 54% reduction of telemedicine electrocardiogram transmission in Genoa telemedicine center (from 364 to 166), 68% in Brescia (from 5.745 to 1.905), 24% in Bari (from 15.825 to 11.716); relative reduction according to electrocardiogram diagnosis was 38% for acute coronary syndrome, 40% for other acute CVD in Genoa center, 24% for acute coronary syndrome, and 38% for other acute CVD in Bari. Male/female ratio remained substantially unchanged. A dramatic reduction of telemedicine access for CVD was observed during Covid-19 outbreak in March 2020 in Italy. The reduction was substantially consistent for all electrocardiogram findings, ACS, other acute CVD and normal.

## Introduction

Severe acute respiratory syndrome coronavirus 2 (SARS-CoV-2) is a positive-sense single-stranded RNA virus, responsible for the ongoing pandemic of Covid-19. The disease was first reported in Wuhan, China, but quickly spread out worldwide, reaching Europe and Italy in late February 2020. As of 8 April 2020, there have been 1,447,466 total confirmed cases of SARS-CoV-2 infection worldwide; the total number of deaths attributed to the virus is 83,471 [1]. The SARS-CoV-2 dramatically impacted the acute and chronic management of cardiovascular disease, accounting from an unexpected drop in urgent referral and hospitalization for acute cardiovascular disease in regions interested by the pandemic in Italy and United States [2].

Telemedicine support is currently widely implemented in clinical practice for the management of cardiovascular disease, both in acute and chronic conditions [3, 4]. Hospital networks for the treatment of ST-elevation acute myocardial infarction (STEMI) are currently based on telemedicine support and remote telemedicine triage with pre-hospital electrocardiogram [5, 6]. Telemedicine is also used for early diagnosis of cardiovascular disease and cardiovascular rehabilitation and chronic heart failure [7–9].

We, therefore, aimed to assess the impact of 2020 SARS-CoV-2 outbreak on telemedicine management of cardiovascular disease in Italy.

## Methods

We analyzed data from three telemedicine dispatch centers, one located in Genoa, serving private clients (pharmacies, general practitioners [10]), another located in Brescia (serving pharmacies), and another located in Bari, serving regional public STEMI network and emergency medical service in Apulia (4 million inhabitants) [11]. Demographic data (age, gender) and principal electrocardiogram diagnosis were collected and analyzed. Records from the time interval March 1, 2020 and April 1, 2020 were compared with the corresponding period in 2019.

### Statistical analysis

Continuous variables were expressed as mean ± standard deviation and compared with Student’s *t* test, categorical variables as percentages and compared with *χ*^2^ test. Normal distribution of variables was assessed with Kolmogorov–Smirnov and Lilliefors test. Changes in percentages were reported with 95% confidence intervals.

A *p* < 0.05 was considered as statistically significant.

## Results

### Non-urgent telemedicine centre (Genoa)

**Fig. 1:**
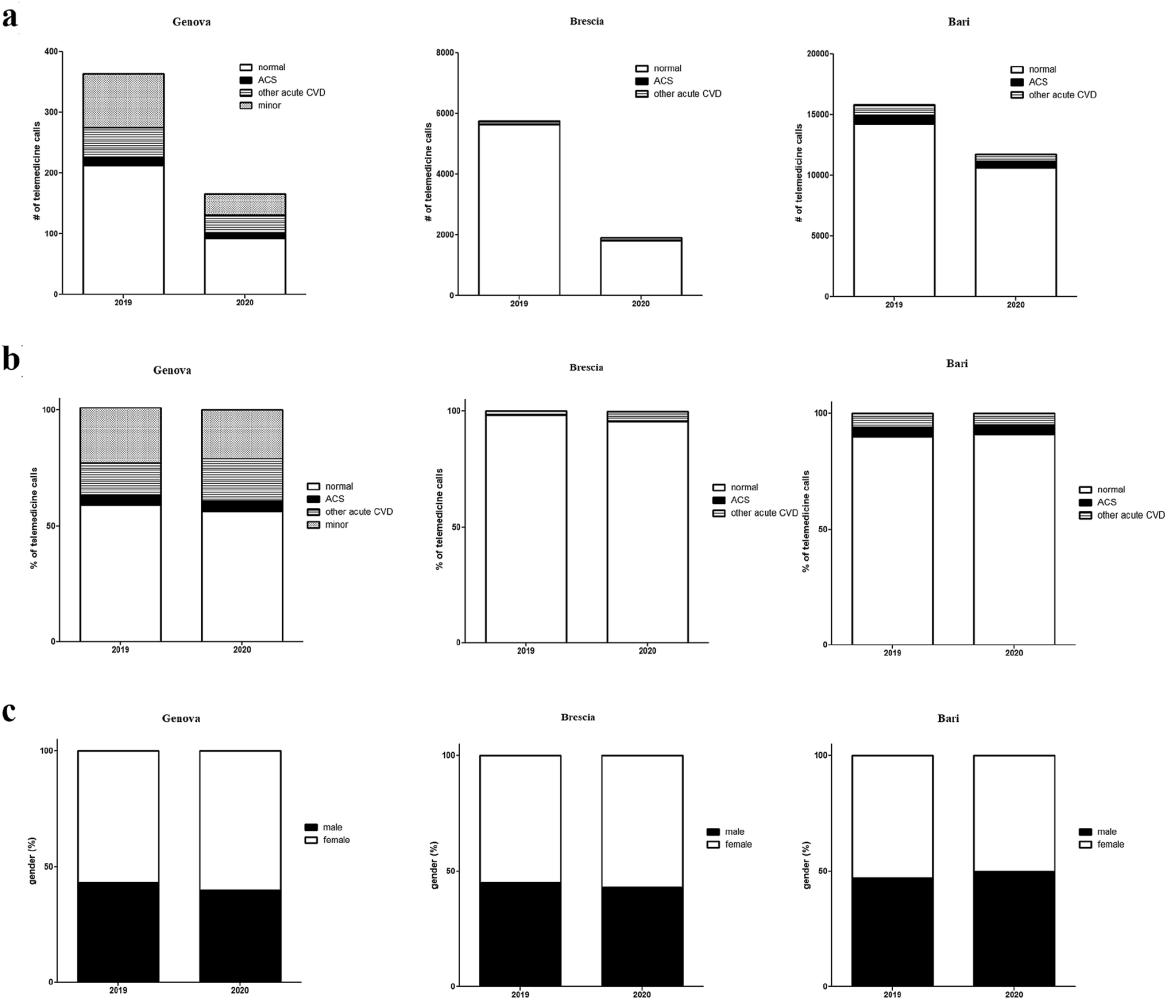
**a** Changes in pre-hospital electrocardiogram number and diagnosis March 2019/March 2020 during Covid-19 outbreak in Italy. A 54% reduction was observed for non-urgent dispatch center in Genoa, 67% in Brescia, and 24% for EMS dispatch center in Bari. The relative reduction of diagnosis with suspect acute coronary syndrome was 24–38%. **b** Comparison of relative prevalence of diagnosis in pre-hospital telemedicine electrocardiograms. **c** Male/female ratio comparison of pre-hospital telemedicine electrocardiograms

The comparative analysis of data shows a 54% (95% CI 49–59%, *p* < 0.001) reduction of telemedicine electrocardiogram transmission from 364 to 166 in the index period (Fig. 1a). Relative reduction according to electrocardiogram diagnosis was 38% (95% CI 7–69%, *p* < 0.05) for acute coronary syndrome (from 13 to 8), 40% (95% CI 26–54%, *p* < 0.001) for other acute CVD (from 50 to 30), 60% (95% CI 50–70%, *p* < 0.001) for minor findings and 56% (95% CI 49–63%, *p* < 0.001) for normal electrocardiogram findings; relative prevalence of different diagnoses remained comparable between 2019 and 2020 (p n.s.) (Fig. 1b). In addition, male/female ratio remained substantially unchanged (40/60, p n.s.) (Fig. 1c). Mean age of patients observed increased from 69 to 73 years (*p* < 0.05).

### Non-urgent telemedicine centre (Brescia)

The comparative analysis of data shows a 67% (95% CI 66–68%, *p* < 0.001) reduction of telemedicine electrocardiogram transmission from 5.745 to 1.905 in the index period (Fig. 1a). Relative reduction according to electrocardiogram diagnosis was 14% (95% CI 7–21%, *p* < 0.001) for other acute CVD (from 92 to 79), and 68% (95% CI 67–69%, *p* < 0.001) for normal electrocardiogram findings (from 5.648 to 1.820); relative prevalence of different diagnoses remained comparable between 2019 and 2020 (p n.s.) (Fig. 1b). In addition, male/female ratio remained substantially unchanged (Fig. 1c). Mean age of patients observed increased from 42 to 47 years (*p* < 0.05).

### Urgencies telemedicine centre (Bari)

The comparative analysis of data shows a 24% (95% CI 23–25%, *p* < 0.001) reduction of telemedicine electrocardiogram transmission from 15.825 to 11.716 in the index period (Fig. 1a). Relative reduction according to electrocardiogram diagnosis was 24% (95% CI 21–27%, *p* < 0.001) for acute coronary syndrome (from 703 to 533, − 170), 38% (95% CI 35–41%, *p* < 0.001) for other acute CVD (from 904 to 558, − 346), 25% (95% CI 24–26%, *p* < 0.001) for other minor or normal electrocardiogram findings (from 14.218 to 10.625, − 3.593); and relative prevalence of different diagnoses remained comparable between 2019 and 2020 (p n.s. for ACS) (Fig. 1b). Male/female ratio remained substantially unchanged (47/53 in 2019, 50/50 2020) (Fig. 1c).

## Discussion

To the best of our knowledge, this is one of the first reports showing the dramatic impact of Covid-19 on telemedicine management of patients with CVD. The number of patients examined by remote electrocardiogram was more than halved for non-emergency calls and reduced by one-fourth for emergency calls, with unpredictable consequences in terms of outcome and survival of patients with CVD.

SARS-CoV-2 infection may cause severe acute respiratory syndrome, but extra-pulmonary symptoms may also be present, such as cardiovascular, neurological and gastroenterological manifestations; endothelial cell dysfunction and impaired microcirculatory function contribute markedly to life-threatening complications of Covid-19, such as venous thromboembolic disease and multiple organ involvement. Patients with Covid-19 infection may show distinctive vascular features, consisting of severe endothelial injury associated with the presence of intracellular virus and disrupted cell membranes, widespread thrombosis with microangiopathy, and alveolar capillary microthrombi [12]. Severe cases of Covid-19 may be characterized by dramatic microvascular injury syndrome mediated by activation of complement pathways and an associated procoagulant state [13].

SARS-CoV-2 infection not only is linked to CV consequences and higher rates of worse outcome in patients with CVD, but may also dramatically compromise the management of such patients. Diabetic population, for instance, is known to have the highest CV risk and therefore deserves targeted therapeutic management and adequate glycemic control; the Covid-19 pandemic may have also led to a reduction in the medical management, even in telemedicine, of patients with diabetes. This aspect could certainly have influenced the outcome of these patients in particular for acute CV events [14].

Even though the larger part of missed patients, in absolute terms, was represented presumably by subjects with expected normal electrocardiogram findings, 24–40% of patients with an expected electrocardiogram diagnosis of ACS or other acute CVD were lost without a diagnosis. In absolute terms, more than 500 patients with acute CVD were not treated through recommended networks and within recommended times in 1 month in Apulia. Patients lost were relatively even more in the smaller population from Genoa telemedicine network. The outcome of such “lost” patients could not be exactly defined, but should be presumably worse.

Several hypotheses have been proposed to explain such dramatic apparent reduction of CVD burden during Covid-19 outbreak. The fear of contagion and lockdown government prescriptions may have discouraged many subjects with CV symptoms from seeking medical and specialistic cardiologic advice.

According to other hypotheses, lower pollution levels because of traffic reduction may be partly responsible for heart attack reduction [15]. Data from large meta-analysis study show that all the main air pollutants, with the exception of ozone, were significantly associated with an increase in myocardial infarction risk (carbon monoxide, nitrogen dioxide, sulfur dioxide, PM(10), and PM(2.5) [16].

Other authors explain apparent reduction in incidence of acute CVD with altruistic reasons; patients may consider their CV symptoms as not really relevant in comparison to critical conditions affecting Covid-19 patients and renounce seeking for medical assistance. Subjects in over-stressing situations may tend to delay presentation to hospital in case of acute coronary symptoms [17]. Stress condition has been shown as a factor responsible for delayed medical seek and emergency department presentation in patients with ACS [18]. Very recently, Greco et al. suggest that pandemic‐derived stress may be responsible for the behavior of ACS patients, influencing a propensity to delay the call for help [19]; patients who later accessed to care were often unaware of their delaying behaviors because pandemic‐related stress seemed to play at unconscious level, largely hampering patients’ self‐perception capability.

It has been hypothesized that symptom overlap between stress and CVD (i.e., tachycardia, dyspnea, diaphoresis) may be confusing to patients in stressing conditions, leading them to discount the possibility of cardiac problems and subsequently delay medical evaluation. In case of previous individual CV events, history may contribute to fear and perceived helplessness regarding a new CV event during a catastrophe, and that these emotions may prevent from accessing care [20].

This increased burden of non-communicable disease, however, alters the vulnerability of populations to disaster as persons with chronic disease may be more greatly impacted by crippling of healthcare system that results from large-scale emergencies [21]. Noteworthy, the occurrence of CVD complications usually peaks after catastrophic events [22]. Unexpectedly, instead, the apparent burden of CVD, at least as considered from a telemedicine perspective, appears reduced during the present Covid-19 outbreak in Italy.

For sure, lower access rates of subjects with acute CVD in Covid-19 outbreak are confirmed also for telemedicine cardiology. Telemedicine, and its applications in the field of CVD, may faithfully mirror all those contingent disturbing situations that can influence the conditions of patients with CVD. Previous studies showed that occasional hype in mass media and press may negatively impact annual rates of influenza vaccination, with consequent dramatic impact on emergency service workloads supported by telemedicine [23]. Catastrophic events may also translate into peaks in emergency and telemedicine calls and stressed emergency services [24].

The paradox is represented by the fact that telemedicine support, which would be extremely useful in the management of patients with CVD, both in the pre-hospital setting and during emergency room access and hospital stay, even in extra-ordinary situations such as catastrophes, is itself crippled by the current pandemic.

Presumably, the lack of generalized implementation of telemedicine assistance for patients with CVD and of widely known telemedicine initiatives of chronic support for CVD patients may be responsible for such underutilization of telemedicine support, just when needed most.

This transient eclipse of CVD and CVD patients surely warrants for the next future a radical reconsideration of current policies and strategies of implementation of telemedicine support for the management of CVD, which evidently failed in their task at the most critical moment. A call for action and urgent initiatives aimed at implementing a larger use of telemedicine just in the case of catastrophic events such as Covid-19 pandemic, when telemedicine support would result extremely useful, are surely warranted.

## Conclusions

A dramatic reduction of telemedicine access for CVD was observed during Covid-19 outbreak in March 2020 in Italy. The reduction of telemedicine support was consistent for all electrocardiogram findings, ACS, other acute CVD and normal. A call for action promoting a larger utilization of telemedicine support is warranted, particularly in the case of pandemics, when telemedicine support would result extremely useful.

## Limitations

Main limitation of the study is the absent confirmation of first telemedicine diagnosis based on pre-hospital electrocardiogram after hospital admission; presumably, small rates of false positive or false negative should be assumed. However, rates of false positive and false negative could be assumed as comparable between 2019 and 2020.

Data on age of patients from one telemedicine center are also not available. Clinical and therapeutic data from patients are also lacking.

Dispatch centers included in the study are very different (private clients vs public health system, non-urgent telemedicine with less transmissions vs urgency telemedicine center with a relevant transmission of data), but such differences, far from representing a mere limitation, may provide data from a more complete real-world scenario.
